# Cost-Impact Analysis of a Novel Diagnostic Test to Assess Community-Acquired Pneumonia Etiology in the Emergency Department Setting: A Multi-Country European Study

**DOI:** 10.3390/ijerph20053853

**Published:** 2023-02-21

**Authors:** Hirad Houshmand, Camilla Porta, Lorenzo Pradelli, Matteo Pinciroli, Giovanni Sotgiu

**Affiliations:** 1 DiaSorin SpA, Via Crescentino, 13040 Saluggia, Italy; 2 AdRes Health Economics and Outcomes Research, 10121 Torino, Italy; 3 Scienze Mediche Chirurgiche E Sperimentali, Università degli Studi di Sassari, 07100 Sassari, Italy

**Keywords:** community-acquired pneumonia, diagnostic testing, lower respiratory tract infection, cost-impact, host-response

## Abstract

Background: We aimed to estimate the economic and clinical impacts of a novel diagnostic test called LIAISON^®^ MeMed BV^®^ (LMMBV), which can differentiate bacterial from viral infections, in patients with community-acquired pneumonia (CAP) in emergency departments. Methods: A cost-impact simulation model was developed to investigate the financial consequences of the introduction of LMMBV into the standard of care (SOC) diagnostic process in Italy, Germany, and Spain. Clinical outcomes were expressed as antibiotic patients and days saved, reduced hospital admissions, and shortened hospital length of stay (LOS). Cost savings were evaluated from the perspectives of third-party payers and hospitals. A deterministic sensitivity analysis (DSA) was carried out. Results: LMMBV was associated with a reduction in antibiotic prescriptions, treatment duration, and LOS. Furthermore, the adoption of LMMBV would allow savings per patient up to EUR 364 and EUR 328 for hospitals and EUR 91 and EUR 59 for payers in Italy and Germany, respectively. In Spain, average savings per patient could reach up to EUR 165 for both payers and hospitals. Savings were most sensitive to test accuracy, with DSA confirming the robustness of the results. Conclusions: Combining LMMBV with the current SOC diagnostic process is expected to provide clinical and economic benefits in Italy, Germany, and Spain.

## 1. Introduction

Community-acquired pneumonia (CAP) is one of the most important causes of morbidity and mortality (2.4 million deaths yearly, the fourth leading cause) worldwide [1,2]. In the European Union (EU), the diagnosis of incident cases is high: 9.7, 4.63, and 2.93–3.06 per 1000 person-year in Germany, Spain, and Italy, respectively [3,4,5,6,7]. Its clinical and economic burdens are expanding with the increased proportion of elderly people and/or comorbidities in the general population [8,9]. In Europe, the estimated annual healthcare cost is higher than EUR 10 billion, with EUR 5.7, EUR 0.5, EUR 0.2, and EUR 3.6 billion for inpatient care, outpatient care, medications, and indirect costs, respectively [6].

The etiology of CAP is not defined in 62% of cases [10], and in the three above-mentioned EU countries, the rates of unidentified pathogens are high [11]. Early detection can improve the quality of therapeutic prescription, decreasing direct and indirect costs. No rapid diagnostic tests can discriminate viral origin to avoid relying on an empirical approach [12]. It was estimated that up to 34.8% of antibiotic days were unnecessary [13], increasing the risk of antimicrobial resistance (AMR) and adverse events (AE).

A new host-response diagnostic test, called LIAISON^®^ MeMed BV^®^ (LMMBV), was developed to differentiate bacterial from viral pathogens. As the first high-throughput and high-quality chemiluminescence immunoassay (CLIA) test, it measures the serum levels of three key host-immune proteins: tumor necrosis factor-related apoptosis-inducing-ligand (TRAIL), interferon gamma-induced protein 10 (IP-10), and C-reactive protein (CRP). Their levels are integrated with a powerful machine learning algorithm into a qualitative score in less than an hour. A score of 0–35 indicates a viral origin, whereas a score of 65–100 indicates a bacterial etiology; the range of 35–65 is considered indeterminate, and other diagnostic tests are needed [14].

A cost-impact model was developed that aimed to evaluate the economic impact of LMMBV uptake in Italy, Germany, and Spain from third-party payer and hospital perspectives. Clinical and economic outcomes associated with treatment guided by standard of care (SOC) diagnostics and treatment guided by SOC combined with LMMBV (SOC + LMMBV) in adult CAP patients in the emergency department (ED) were compared. This paper is an adaptation of a previously published analysis carried out for the United States [10].

## 2. Materials and Methods

### 2.1. Model Overview

A cost-impact model was developed in Microsoft Excel to assess the economic impact and clinical benefit of SOC diagnostics combined with LMMBV vs. SOC alone. A hypothetical cohort of 1000 adult patients with symptoms consistent with CAP in the ED was considered in four scenarios (Table 1). The main analysis focused on the clinical and economic impacts of differences in antibiotic stewardship and their healthcare implications. The other scenarios combined the influence of LMMBV on antibiotic prescriptions with impacts on hospital admission rates (scenario 1), hospital length of stay (LOS) and diagnosis-related group (DRG) reallocation (scenario 2), and hospital admission rates, hospital LOS, and DRG reallocation in combination (scenario 3).

### 2.2. Model Structure

**Figure 1:**
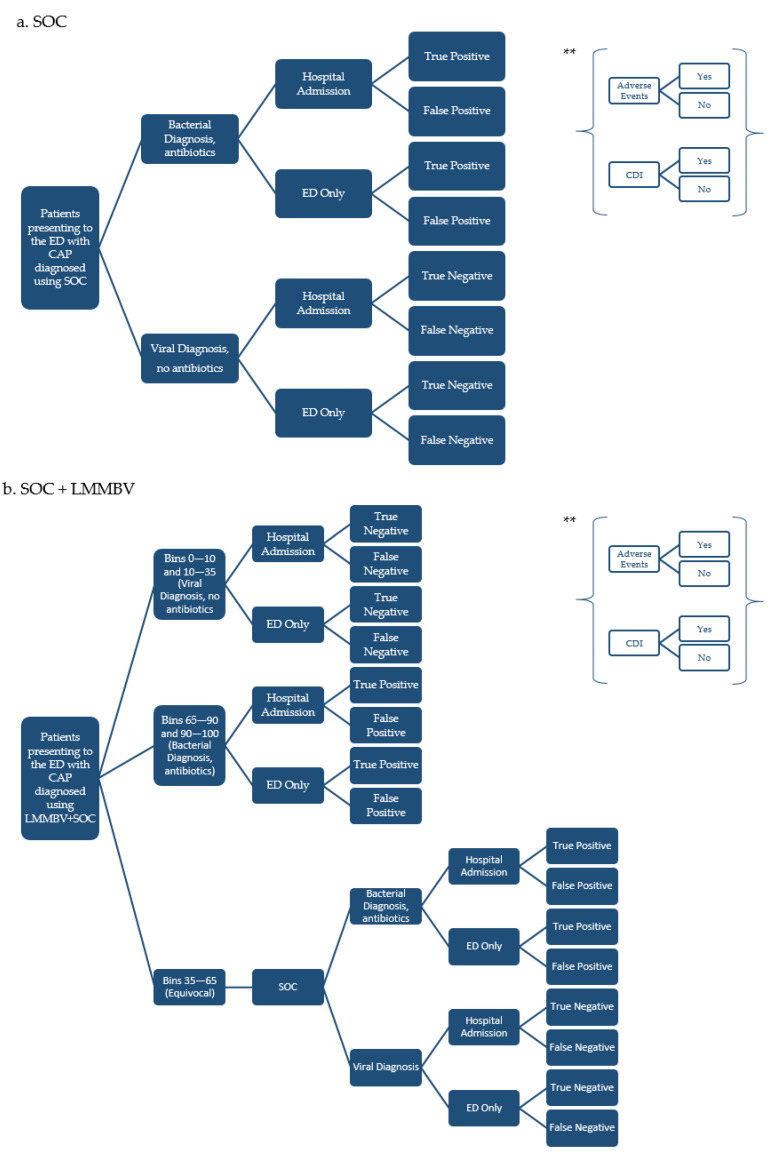
Simplified model scheme [10]. ** At each leaf node of the decision tree, patients may experience antibiotic-related AEs or CDIs.

Based on the scientific literature, clinical outcomes were simulated according to a decision tree model, presenting a mathematical simplification of possible clinical pathways experienced by suspected CAP patients in the ED (Figure 1).

In the first treatment arm, patients receive a bacterial or viral diagnosis based on SOC diagnostic processes (i.e., X-ray, complete blood count, and viral PCR testing). Subsequently, patients are either admitted to the hospital or treated in the ED. They are then classified according to the accuracy of the diagnosis as a true positive (TP; bacterial diagnosis), false positive (FP; misclassified viral etiology), true negative (TN; viral diagnosis), or false negative (FN; misclassified bacterial etiology) [10].

SOC + LMMBV-diagnosed patients follow the same pathway, which is informed by the test results. With a bacterial diagnosis (LMMBV test scores from 65–100), patients are administered antibiotics. In the case of a viral diagnosis (scores from 0–35), the patient is not exposed to antibiotics. Patients with scores from 35–65 follow the SOC-guided treatment path.

Early and appropriate therapy can improve clinical outcomes, reducing the risk of adverse events and *Clostridium difficile* infections (CDIs). On the other hand, FP patients would undergo unnecessary antibiotic treatment, and FN patients would remain in treatment for a longer duration due to their worsening clinical condition.

Patients were stratified into four groups according to their pneumonia severity index (PSI 1 and 2, PSI 3, PSI 4, PSI 5).

### 2.3. Sensitivity Analysis

A one-way deterministic sensitivity analysis (DSA) was performed to assess the effect of uncertainty with input parameters on cost-impact results. These parameters were varied, one at a time, between the lower and upper limits of 95% confidence intervals (if available) or ±20% of the base case analysis, while all the others were held stable. Since the data related to LMMBV specificity and sensitivity were based on an ongoing study, the related confidence intervals were not available at the time of this analysis. However, based on similar publications [15,16,17,18,19], it was considered appropriate and more plausible to vary both LMMBV and SOC test accuracy between values corresponding to ±5% of the base case.

### 2.4. Inputs

#### 2.4.1. Clinical Inputs

The baseline clinical inputs are summarized in Table 2 and Table 3 [10].

To model clinical decision making following the test results, the physician’s decision to administer antibiotics was assumed to be a function of both bacterial/viral diagnosis and pneumonia severity. In this hypothesis, physicians would follow diagnostic test results 100%, 80%, 50%, and 0% of the time for PSI scores 1 and 2, 3, 4, and 5, respectively; all other cases would be associated with the administration of antibiotics. In addition, hospital admission rate was based on disease severity.

The mean antibiotic treatment duration for TP and FP patients was 5 days [12]. FN patients were assumed to be exposed to antibiotics for a 50% longer duration compared to TP and FP patients (7.5 days) due to disease progression because of antibiotic delays.

SOC patients were assumed to be administered prediagnostic inpatient antibiotics for a mean duration of 1.5 days. FP and TP patients would continue treatment for a further 3.5 days, whereas FN patients would re-start therapy for an average overall duration of 7.5 + 1.5 days. On the contrary, it was assumed that patients in the SOC + LMMBV arm would avoid prediagnostic antibiotic prescription, considering its short diagnostic turnaround time.

The probability of experiencing adverse events varies based on treatment duration, using a treatment-related AE rate of 0.002 per antibiotic day [27]. The risk of CDI was calculated by applying a 3.24% probability of infection in the SOC arm and a hazard ratio of 1.09 in the SOC + LMMBV arm (for each additional day of antibiotic treatment) [28].

Country-specific LOS values were retrieved from the literature. Length of stay for each PSI risk class in Germany was obtained by re-proportioning the mean length of 9 days, as in Ostermann et al. [29].

**Table 3:** Baseline clinical input: country adaptation.

	Value			Ref		
	Italy	Germany	Spain	IT	GE	SP
Baseline antibiotic treatment days (TP and FP patients)	5			[12]		
Antibiotic treatment days for FN patients	7.5			Assumption		
Baseline hospital LOS for hospitalized patients (days)						
PSI score 1 and 2	4.74	7.04	2.56	[30]	Assumption [29]	[31]
PSI score 3	8.71	7.80	5.39			
PSI score 4	8.09	9.01	8.82			
PSI score 5	11.76	12.15	10.22			

#### 2.4.2. Costs

According to the third-party payer and hospital perspectives adopted, only direct healthcare costs were considered, including diagnostic testing and ED visit, antibiotic administration, adverse events/CDI management, and hospitalization (Table 4). Cost items are not mutually exclusive since both payers and hospitals may incur the same cost, albeit generally at a different cost level. However, different reimbursement systems apply depending on the country: in Italy and Germany, the national payment system is based on the DRG tariff or Germany-DRG (G-DRG), whereas in Spain, the National Health System (NHS) fully reimburses hospitals via a global budget principle. Therefore, only one perspective is considered for the Spanish setting since the hospital component of NHS healthcare expenditures generally corresponds to actual hospital costs (the two perspectives coincide). Costs were estimated by multiplying resource use, obtained from the model, by the unit cost of each resource (Table 5). Costs were expressed in euros (EUR) and updated to 2022.

Inpatient and outpatient antibiotic costs per day were calculated by averaging the daily cost of antibiotics recommended by international guidelines [12]. The per-day cost was computed by combining the net ex-factory price and posology [12,40,41,42]. Regarding intravenous (IV) drugs, the cost of administration was retrieved from the scientific literature for Italy and from outpatient tariffs for Germany. No data related to the cost of IV administration in Spain were retrieved. Hence, its unit cost was estimated based on Giusti et al. [43] and adjusted to the Spanish context through a health services-specific cross-country price index reported by Lorenzoni et al. [44].

Hospitalization costs were estimated by multiplying the bed-day cost [29,45,46] by the length of stay. The costs of AEs and CDIs were quantified in additional hospital days, obtained from the related probabilities and specific attributable LOS [25,26].

From a third-party payer perspective (in Italy and Germany), hospitalization costs for a CAP episode were calculated by weighting the national DRG tariffs by the number of discharges (DRG 89/90 in Italy and DRG E79A/E65A/E79C in Germany) [32,45,46]. The cost of outpatient CDIs was inferred by applying the DRG tariff related to CDI (DRG 572 in Italy and G77B in Germany) under the assumption that those antibiotic patients who were not initially admitted to hospital would eventually be hospitalized with a diagnosis associated with CDI (gastrointestinal disease) [32,48].

#### 2.4.3. Scenarios

Scenario 1 implements the potential impact of LMMBV on hospitalization rates. For SOC + LMMBV patients, 20% (PSI 1 and 2), 40% (PSI 3), 30% (PSI 4), and 0% (PSI 5) of hospital admission decisions were assumed to be determined by test outcomes, with bacterial diagnoses associated with hospital admission and viral diagnoses associated with discharge. An additional risk of delayed hospitalization for FN diagnoses in both arms was kept in consideration, assuming that these patients, initially discharged from the ED, would eventually return to receive appropriate treatment.

Scenario 2 included the potential impact of LMMBV on LOS (for hospitals in all countries and payers in Spain) and hospital costs (for payers in Italy and Germany).

From the hospital perspective, a change in LOS was associated with an expected reduction in bacterial-diagnosed and FN hospital admissions, characterized by longer hospital stays. Thus, the LOS for SOC + LMMBV was recalculated considering a different LOS for each diagnostic pathway. Bacterial-diagnosed hospital stays (TP or FP) were assumed to be equal to baseline LOS values, whereas the LOS for TN and FP was calculated assuming a 31% reduction and a 50% increase, respectively, in LOS compared to those related to patients with a bacterial diagnosis [50,51,52]. The mean LOS was calculated as a weighted average according to the expected frequency of each test outcome.

Shorter hospital stays would not benefit Italian and German payers, as they reimburse hospitals via a fixed tariff determined by DRG codes assigned at discharge. More precisely, the baseline hospitalization cost for a CAP episode was calculated as a weighted mean of DRG tariffs related to pneumonia, considering the number of discharges per DRG as weights. In this context, the impact of LMMBV on patient severity was quantified as a reduction in the mean cost of hospitalization through a reallocation of DRG codes. Following an early and accurate diagnosis, LMMBV patients were expected to receive early and appropriate treatment. As a result, LMMBV was assumed to reduce the proportion of more severe cases and increase the number of patients classified as less severe. Thus, a percentage of patients, equal to the difference in false-negative diagnoses between SOC and SOC + LMMBV, was reallocated from the DRG codes related to the most severe conditions to that of a less severe condition, resulting in a mean cost per CAP episode of EUR 2617 and EUR 3104 in Germany and Italy, respectively.

Finally, the third scenario combined the first two, considering the impact of LMMBV on both the hospital admission rate and LOS/DRG reallocation.

## 3. Results

LMMBV showed higher sensitivity and specificity in comparison with the current SOC diagnostic process, allowing for a reduction in bacterial/FN diagnoses and of risk progression and clinical complications. Improvement in the diagnostic accuracy of a suspected CAP would save healthcare resources in terms of antibiotic patients and days, hospital admissions, and hospital LOS (Table 6).

LMMBV is associated with a reduction in antibiotic prescriptions (43%) and in antibiotic days (1.02 per patient). Moreover, 8 hospital admissions per 1000 patients could be avoided with LMMBV, saving more than 300 hospital days.

Savings are relatively low in the main analysis but gradually increase in the scenario analyses when considering impacts on hospital admission decisions and hospital LOS/DRG reallocation (Table 7 and Table 8). The cost of hospital stay was the main driver in the selected settings. From the hospital perspective, LMMBV would result in cost savings per patient in the range of EUR 61–EUR 364 in Italy and EUR 78–EUR 328 in Germany, whereas up to EUR 91 and EUR 59 could be saved for Italian and German payers, respectively. Instead, in Spain, the average savings per patient could reach approximately EUR 165 for both payers and hospitals.

**Figure 2:**
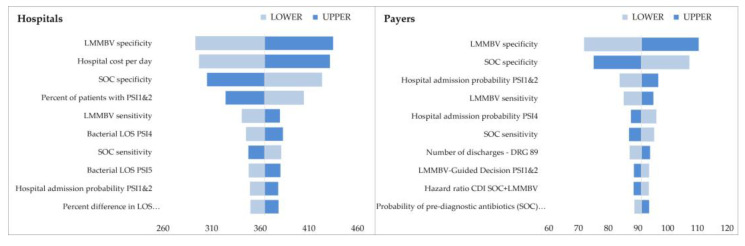
DSA scenario 3—Italy.

**Figure 3:**
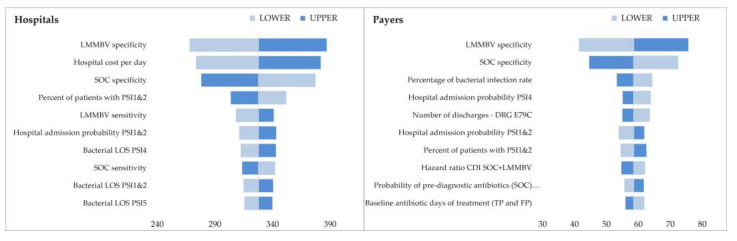
DSA scenario 3—Germany.

**Figure 4:**
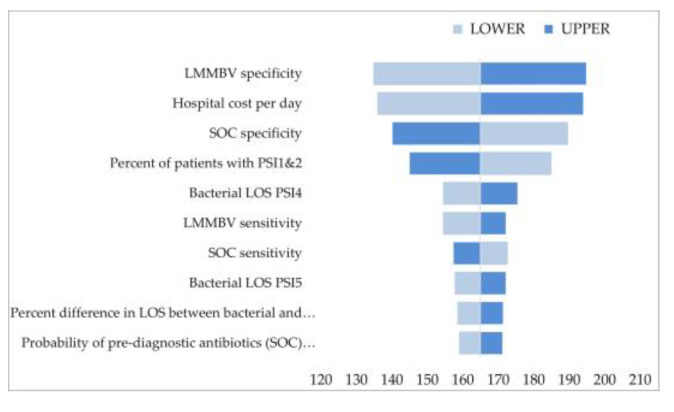
DSA scenario 3—Spain.

The DSA tornado diagrams show the 10 most influential variables in scenario 3 (Figure 2, Figure 3 and Figure 4), while complete results are reported in the supplementary materials (Figures S1–S3). LMMBV and SOC specificity and sensitivity were the variables with the greatest impact on the results. Hospital savings were also sensitive to changes in hospital cost per day. Overall, the results are robust to tested parameter variations, and in all cases, the main conclusions hold valid.

## 4. Discussion

A relevant challenge in the management of CAP patients is making the correct etiological diagnosis. International and national guidelines suggest a combination of different diagnostic approaches, from radiography to blood tests and cultures [12]; however, no gold standard exists, and appropriate diagnostic modalities/approaches for an accurate and rapid CAP etiological diagnosis are currently an unmet need [53].

This study estimates the economic impact of the inclusion of a novel host-response diagnostic test in the current SOC process for patients with suspected CAP in the ED. Owing to its better sensitivity and specificity compared to SOC, combined with its timeliness, LMMBV would reduce antibiotic overuse, saving approximately 1020 antibiotic days per 1000 patients and lowering the risk of clinical complications. Thus, combining LMMBV with current SOC diagnostic processes is associated with substantial clinical advantages, resulting in considerable savings for both payers and hospitals in different geographic settings. In addition, if the analysis includes a societal perspective, LMMBV economic benefits could increase if other potential savings related to indirect costs (e.g., loss of productivity of both patients and caregivers) are taken into account.

CAP patients can be affected by overprescription of antibiotics, thus contributing to the development of antimicrobial resistance [53]. AMR is a global public health threat. In the European Union, infections from antibiotic-resistant pathogens are associated with more than 30,000 deaths and an annual healthcare expenditure of approximately EUR 1.5 billion [54]. Although the model does not directly quantify the cost savings related to the occurrence and spread of AMR, it shows that LMMBV could potentially reduce antibiotic overuse and misuse, with the positive outcome of reducing the risk of drug-resistant strains being selected and its clinical consequences.

This study is an adaptation of a previously published US model in the context of three European countries [10]. Although several technical similarities can be found, some differences should be highlighted. For each single country, local country parameters (e.g., average length of stay, duration of antibiotic therapy, and applicability of PSI in the country) were selected following the opinions of local experts and scientific data. Clinical inputs were included in the model together with local unit costs. Costs were estimated according to the settings of each national health system.

The selected countries were the most representative; they showed the highest rates of CAP in which the pathogen was not diagnosed [11]. This could be a condition for the implementation of rapid diagnostic tests; however, their adoption in clinical practice or in low-income countries where epidemiological data are scarce [1] is currently unknown.

Three main study limitations should be acknowledged. First, it is a cohort simulation of real-life pathways, so the model cannot include interindividual variations and heterogeneities among clinical practices in different settings. In addition, the model does not take into account subgroups of patients (e.g., the elderly or infants) because of missing data, thus lowering the accuracy of the estimates. Some assumptions and approximations were made when data were not available. Therefore, the uncertainty of input parameters was evaluated in sensitivity analyses.

## 5. Conclusions

Based on the heavy burden of CAP, the inclusion of LMMBV in the diagnostic SOC provides clinical and economic benefits by reducing antibiotic prescriptions, days of therapy, and length of hospital stay.

The model is not a comprehensive health technology assessment (HTA), but it provides clinicians and policy makers with helpful cost estimates. Therefore, it could support decision making and encourage evidence-based recommendations.

## Figures and Tables

**Table 1:** Scenario analysis.

Scenario	Impact of Antibiotic Prescription	Impact of Hospital Admission	Impact of Hospital LOS
Main analysis—Impact of antibiotic prescription only	x		
Scenario 1—Hospital admission rate impact	x	x	
Scenario 2—Length of stay impact/DRG reallocation	x		x
Scenario 3—Reduced hospitalization and length of stay impact/DRG reallocation	x	x	x

**Table 2:** Baseline clinical inputs [10].

	Value		Ref	
	SOC	SOC + LMMBV	SOC	SOC + LMMBV
Bacterial infection rate	37.8%		[20]	
Viral infection rate	62.2%		[20]	
Sensitivity	66.6%	96.7%	[21,22]	[15,16,17,18,19,23]
Specificity	78.3%	89.8%		
Test equivocal rate	NA	8.3%	NA	
Probability of prediagnostic antibiotics				
PSI score 1 and 2	75.0%	NA	Assumption	NA
PSI score 3	90.0%	NA		
PSI score 4	100.0%	NA		
PSI score 5	100.0%	NA		
Prediagnostic antibiotic treatment days	1.5	NA	Assumption	NA
Baseline cohort PSI severity distributions				
PSI score 1 and 2	55.0%		[24]	
PSI score 3	14.0%			
PSI score 4	21.0%			
PSI score 5	10.0%			
Hospital admission probability from the ED				
PSI score 1 and 2	33.5%		[24]	
PSI score 3	78.0%			
PSI score 4	92.0%			
PSI score 5	100.0%			
Probability of antibiotic adverse events				
PSI score 1 and 2	0.65%	0.42%	(c)	
PSI score 3	0.72%	0.52%		
PSI score 4	0.80%	0.66%		
PSI score 5	0.91%	0.91%		
Antibiotic adverse event-attributable LOS	1.3		[25]	
Probability of CDI				
PSI score 1 and 2	2.43%	1.71%	(c)	
PSI score 3	2.92%	2.28%		
PSI score 4	3.24%	2.93%		
PSI score 5	3.24%	3.24%		
Hospital-onset CDI attributable LOS (days)	7.8		[26]	
Community-onset CDI attributable LOS (days)	5.7		[26]	

**Table 4:** Cost drivers by perspective.

Cost Drivers	Hospitals		Payers		Hospitals/Payers
	Italy	Germany	Italy	Germany	Spain
Diagnostic testing	x	x	x	x	x
ED visit	x	x	x	x	x
Inpatient days of antibiotic (AB) treatment	x	x			x
Outpatient days of AB treatment			x	x	x
Adverse Events	x	x			x
Outpatient CDI	x	x	x	x	x
Baseline hospital stay and inpatient CDI	x	x	x	x	x

Outpatient rates and the scientific literature were used to estimate the cost of diagnostic testing and ED visits [32,33,34,35,36,37,38,39]. In Italy, the cost of viral PCR was based on data provided by DiaSorin’s Market Intelligence. The model omits the cost of LMMBV itself.

**Table 5:** Unit costs (EUR).

Economic Inputs	Italy	Ref	Germany	Ref	Spain	Ref
Diagnostic test and ED stay cost						
X-ray	15.5	Tariff 87.44.1 [32]	9.2	Tariff 34240 [34]	9.2	[35]
Complete blood count (CBC)	3.2	Tariff 90.62.2 [32]	1.1	Tariff 32122 [34]	6.7	[35]
Viral PCR	118.5	[37]	85.0	Tariff 32851 [34]	75.0	[36]
ED visit	248.2	[33]	588.1	[38]	236.1	[39]
Antibiotic treatment						
Cost of inpatient AB treatment per day	25.0	Elaboration [12,40]	52.7	Elaboration [12,41]	18.4	Elaboration [12,42]
Including cost per IV administration	6.4	* [43]	7.6	Tariff 02100 [34]	5.6	[43,44]
Cost of outpatient AB treatment per day	1.3	Elaboration [12,40]	5.5	Elaboration [12,41]	1.9	Elaboration [12,42]
Hospital stays—providers						
Hospital cost per day	962.6	[45]	697.2	[29]	488.7	[46]
Hospital stays—payers						
Hospital cost per episode—CAP	3198.8	DRG 89/90 ** [32,47]	2657.7	DRG E79A, E65A, E79C ** [48,49]	-	
Hospital cost per episode—inpatient CDI	3558.0	DRG 89 [32]	4059.0	DRG E79A [48]	-	
Hospital cost per episode—outpatient CDI	3484.0	DRG 572 [32]	3798.0	DRG G77B [48]	-	

* Assuming 5 min of a doctor’s time devoted to infusion therapy; IV, intravenous; ** Weighted for discharges.

**Table 6:** Clinical outcomes per patient—SOC + LMMBV vs. SOC.

	Main Analysis		Scenario 1		Scenario 2		Scenario 3	
	Per Patient	Per 1000	Per Patient	Per 1000	Per Patient	Per 1000	Per Patient	Per 1000
Antibiotic patients avoided	0.43	429	0.43	429	0.43	429	0.43	429
Antibiotic days saved	1.02	1020	1.02	1020	1.02	1020	1.02	1020
Hospital admissions avoided	-	-	0.01	8	-	-	0.01	8
Hospital days saved								
Italy	0.04	38	0.08	82	0.30	303	0.35	351
Germany	0.04	38	0.10	100	0.32	323	0.39	389
Spain	0.04	38	0.06	62	0.27	271	0.30	297

**Table 7:** Savings per patient (EUR).

(A) Italy	Main Analysis		Scenario 1		Scenario 2		Scenario 3	
	Hospital	Payer	Hospital	Payer	Hospital	Payer	Hospital	Payer
Diagnostic testing	-	-	-	-	-	-	-	-
ED visit	-	-	-	-	-	-	-	-
Inpatient days of AB treatment	24.6	-	26.9	-	24.6	-	26.9	-
Outpatient days of AB treatment	-	0.0	-	(0.1)	-	0.0	-	(0.1)
Adverse events	1.1	-	1.2	-	1.1	-	1.2	-
Outpatient CDI	15.9	10.1	20.7	9.2	15.9	10.1	20.7	9.2
Baseline Hospital Stay and Inpatient CDI	19.7	0.9	57.0	26.7	274.8	55.1	315.6	82.1
Total	61.4	11.1	105.8	35.8	316.5	65.3	364.4	91.2
**(B) Germany**	**Main Analysis**		**Scenario 1**		**Scenario 2**		**Scenario 3**	
	**Hospital**	**Payer**	**Hospital**	**Payer**	**Hospital**	**Payer**	**Hospital**	**Payer**
Diagnostic testing	-	-	-	-	-	-	-	-
ED visit	-	-	-	-	-	-	-	-
Inpatient days of AB treatment	51.8	-	56.7	-	51.8	-	56.7	-
Outpatient days of AB treatment	-	0.2	-	(0.3)	-	0.2	-	(0.3)
Adverse events	0.8	-	0.9	-	0.8	-	0.9	-
Outpatient CDI	11.5	11.0	15.0	10.0	11.5	11.0	15.0	10.0
Baseline Hospital Stay and Inpatient CDI	14.3	3.7	54.2	25.4	212.5	26.6	255.3	48.9
Total	78.4	14.9	126.7	35.1	276.7	37.8	327.8	58.5
**(C) Spain (Payers/Hospitals)**	**Main Analysis**		**Scenario 1**		**Scenario 2**		**Scenario 3**	
Diagnostic testing	-		-		-		-	
ED visit	-		-		-		-	
Inpatient days of AB treatment	18.1		19.7		18.1		19.7	
Outpatient days of AB treatment	0.1		(0.1)		0.1		(0.1)	
Adverse events	0.6		0.6		0.6		0.6	
Outpatient CDI	8.1		10.5		8.1		10.5	
Baseline Hospital Stay and Inpatient CDI	10.0		19.9		123.8		134.2	
Total	36.8		49.8		150.5		164.9	

**Table 8:** Total savings (EUR)—1000 patients.

Scenarios	Italy		Germany		Spain
	Hospital	Payer	Hospital	Payer	Hospital/Payer
Main analysis	61,385	11,077	78,437	14,883	36,787
Scenario 1	105,830	35,817	126,731	35,079	49,770
Scenario 2	316,459	65,266	276,685	37,846	150,535
Scenario 3	364,403	91,158	327,806	58,530	164,931

## Data Availability

Not applicable.

## Supplementary Materials

The following supporting information can be downloaded at: https://www.mdpi.com/article/10.3390/ijerph20053853/s1, Figure S1: DSA results—Italy; Figure S2: DSA results—Germany; Figure S3: DSA results—Spain.
