# Supplementary figures and images for: Cost-Impact Analysis of a Novel Diagnostic Test to Assess Community-Acquired Pneumonia Etiology in the Emergency Department Setting: A Multi-Country European Study
Source: Int J Environ Res Public Health. 2023 Feb 21;20(5):3853. doi: 10.3390/ijerph20053853 (PMC10001249; doi:10.3390/ijerph20053853)

Figure S1: DSA results—Italy

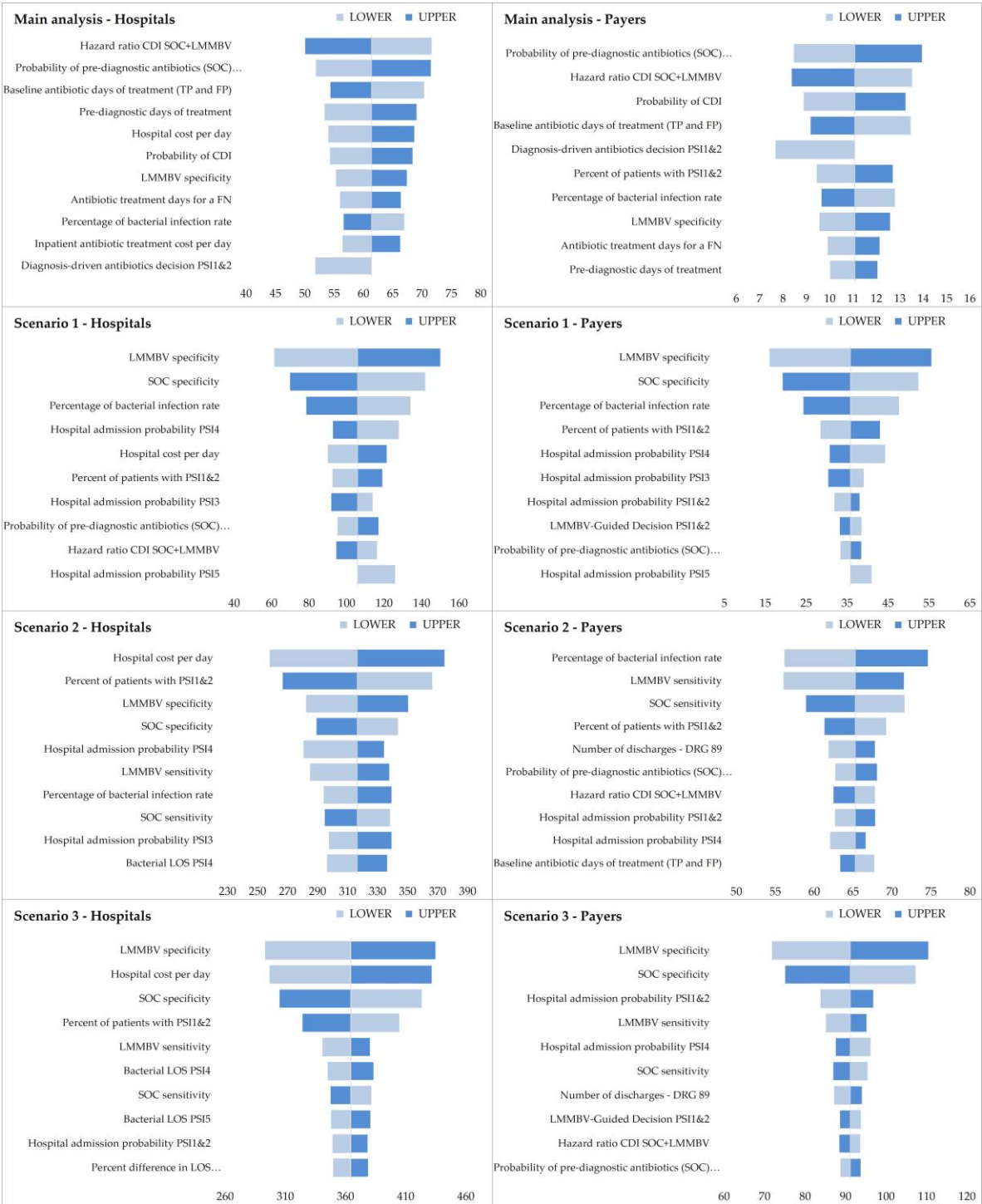

Figure S2: DSA results—Germany

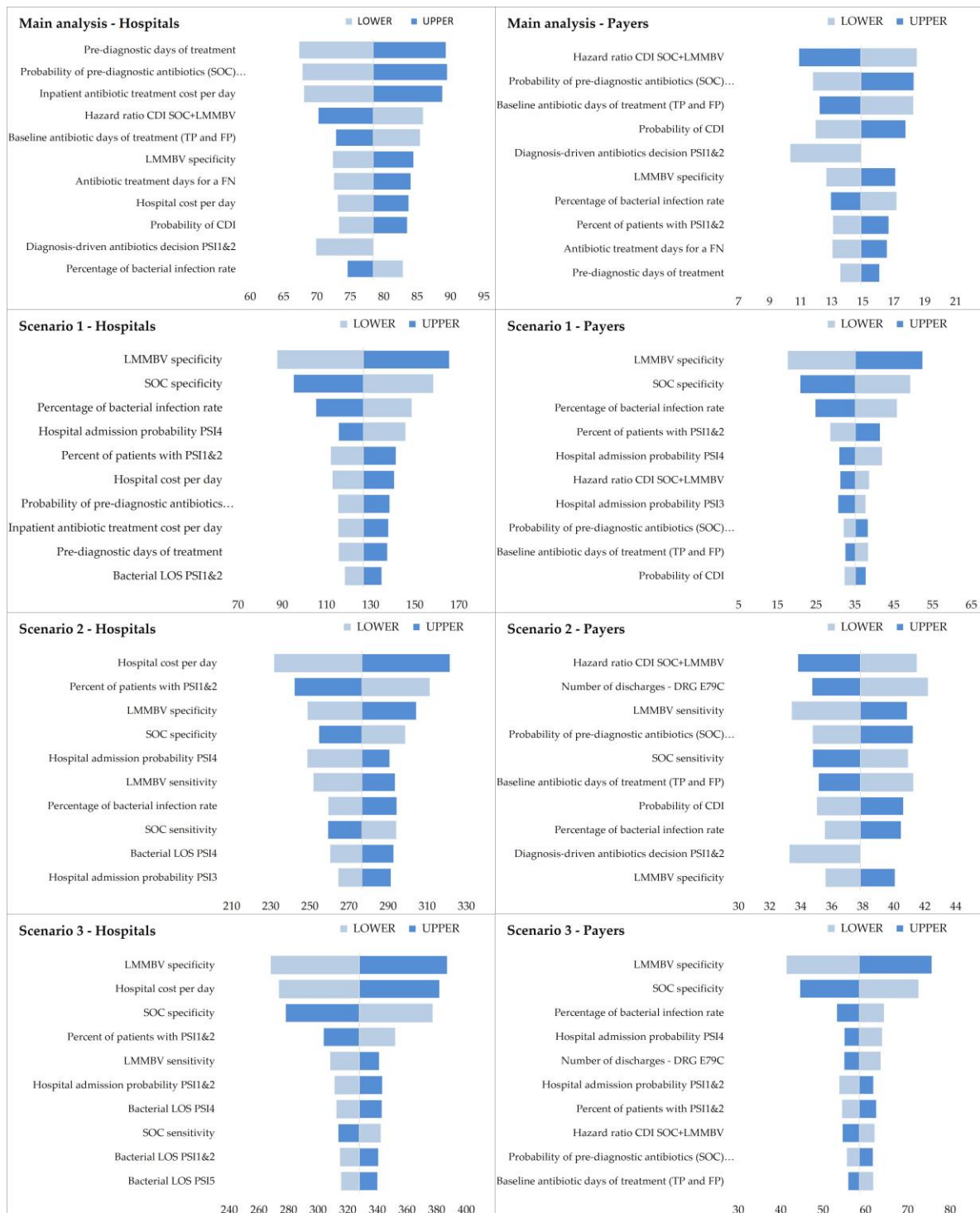

Figure S3: DSA results—Spain

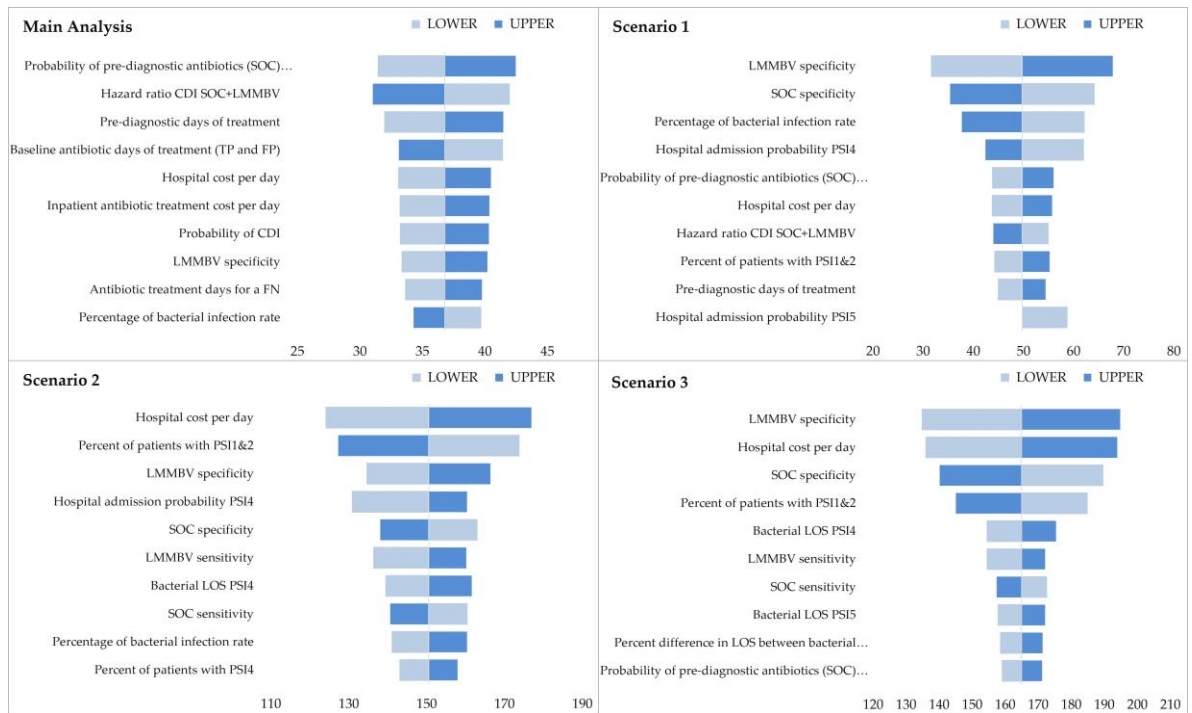

Supplement: Supplementary file 1 [file ijerph-20-03853-s001.zip › ijerph-2111654-supplementary.pdf]
